# The auditory trap: early semantic conflict and late monitoring breakdown drive false memories in cognitive aging

**DOI:** 10.3389/fpsyg.2026.1830028

**Published:** 2026-07-02

**Authors:** Zhichun Zhao, Ting Guo, Jun Fan, Zijie Wang, Jinfeng Huang

**Affiliations:** 1 School of Mathematical Sciences, Faculty of Science and Engineering, University of Nottingham Ningbo China, Ningbo, China; 2 Research Center for Language and Cognition, Ningbo University of Technology, Ningbo, China; 3 Guangdong Key Laboratory of Biomedical Measurements and Ultrasound Imaging, School of Biomedical Engineering, Shenzhen University Medical School, Shenzhen University, Shenzhen, China

**Keywords:** cognitive aging, cross-modal processing, event-related potentials, false memory, source monitoring

## Abstract

**Introduction:**

Cognitive aging increases vulnerability to false memories, yet the neural mechanisms underlying age-related susceptibility across sensory modalities remain poorly understood. Most previous studies have focused on visual orthographic stimuli, leaving the neural dynamics of auditory false memories largely unexplored.

**Methods:**

To examine whether age-related susceptibility to semantic illusions is modality-specific and to characterize its spatiotemporal neural dynamics, we employed a cross-modal Deese–Roediger–McDermott (DRM) paradigm including Visual and Auditory conditions. Sixty-four-channel electroencephalography (EEG) was recorded from healthy younger and older adults. Event-related potentials (ERPs) were analyzed over frontal (300–500 ms) and parietal (600–800 ms) regions to assess familiarity-related and post-retrieval monitoring processes.

**Results:**

Behaviorally, older adults maintained robust old-lure discrimination accuracy in the Visual condition but showed reduced performance in the Auditory condition, characterized by increased false alarms and prolonged reaction times for correct rejections. Electrophysiologically, visual lures elicited a classic FN400 familiarity effect together with relatively preserved late parietal monitoring responses across both age groups. In contrast, auditory lures elicited an early N400-like effect suggestive of increased semantic conflict processing. While younger adults appeared to recruit late parietal monitoring resources to resolve this auditory conflict, older adults showed attenuated and less organized late parietal monitoring activity.

**Discussion:**

These findings provide preliminary evidence for a modality-dependent vulnerability to false memories in cognitive aging. The results suggest that auditory false memories may arise from increased semantic conflict combined with reduced late-stage monitoring processes, highlighting a potential modality-specific mechanism underlying age-related memory distortions. Pending further replication, these findings may contribute to a better understanding of false-memory vulnerability in older adults.

## Introduction

Episodic memory decline is a hallmark of cognitive aging (Aghjayan et al., 2022; Ballesteros et al., 2024), leaving older adults increasingly vulnerable not only to forgetting but also to generating false memories (McCabe and Smith, 2002). These semantic illusions—remembering events or details that never occurred—pose significant risks in everyday life, particularly in critical contexts such as recalling medical instructions or financial agreements. According to the Source Monitoring Framework, false memories occur when individuals successfully retrieve the general meaning or “gist” of an experience but fail to engage the cognitive control processes required to monitor and attribute specific perceptual details to their correct source (Johnson et al., 1993). While the behavioral phenomenon of age-related susceptibility to false memory is well-documented (Devitt and Foster, 2025), the spatiotemporal neurodynamic underlying how the aging brain succumbs to, or attempts to resist, these semantic illusions remain a critical frontier in cognitive neuroscience. The DRM paradigm is ideally suited to test this framework because it systematically induces gist-based activation while requiring source-level discrimination, thereby revealing where source monitoring fails.

Over the past decades, the DRM paradigm has been extensively utilized to map the neural correlates of false memory (Dehon, 2020; Gallo, 2010; Gallo and Roediger, 2002). EEG studies have reliably dissociated two distinct stages of memory retrieval: an early frontal component (FN400, 300–500 ms) reflecting familiarity-driven semantic activation, and a late parietal component (late positive component or LPC, 600–800 ms) indexing effortful detail recollection and post-retrieval monitoring (Shayesteh et al., 2020; Volz et al., 2019). In behavioral terms, stronger recruitment of this late parietal monitoring system is typically expressed as longer reaction times, reflecting additional verification costs. Evidence suggests that older adults may exhibit an over-reliance on early gist-based familiarity to compensate for decaying late parietal monitoring resources (Zhang et al., 2024). Consequently, typical neurophysiological accounts posit those age-related false memories stem from a generalized degradation of the frontoparietal control network (Dennis et al., 2022), leading to indiscriminate acceptance of semantically related lures (Schacter et al., 1998; Sweeney-Reed et al., 2012).

However, a critical limitation in the current literature is the predominant reliance on visual orthographic stimuli (text), largely overlooking the sensory modality through which semantic information is delivered (Palmiero et al., 2014). Speech and text impose fundamentally different demands on the cognitive system (Ou et al., 2015). Unlike visual text, which provides stable orthographic cues that can be rescanned, auditory speech is inherently transient. Its acoustic-phonetic features decay rapidly from sensory buffers. As these perceptual traces fade before retrieval decisions are finalized, listeners must rely more heavily on predictive semantic reconstruction, which increases early (300–500 ms) prediction error and amplifies N400-like semantic conflict when lures mismatch the expected source. This transient acoustic input therefore places greater demands on working memory and post-retrieval monitoring processes (Lim et al., 2019). Despite this, it remains unclear whether the aging brain’s susceptibility to semantic illusions is domain-general or modality-specific (Failes et al., 2020). Crucially, the dynamic neural interplay, from early semantic conflict detection to late cognitive control, that dictates whether an auditory semantic illusion is successfully suppressed or falsely endorsed in the aging brain has yet to be systematically mapped.

To bridge this gap, the present study investigates the cross-modal neurocognitive dynamics of false memory generation and suppression in cognitive aging. Utilizing a modified DRM paradigm (Baioui et al., 2012; Watson et al., 2004), we compared the behavioral performance and ERPs of healthy older adults and younger adults across Visual and Auditory conditions. By extracting temporally distinct ERP components over pre-defined frontal and parietal regions of interest, we aimed to trace the entire evolution of source monitoring. We hypothesized that older adults may exhibit a modality-specific vulnerability, producing an “auditory trap” pattern, characterized by preserved discrimination in the Visual condition but a reduced performance in the Auditory condition (Edwards et al., 2017). Furthermore, we postulated that this auditory failure would not merely result from a lack of early detection, but rather from a profound exhaustion of late parietal monitoring resources following intense early semantic conflict.

Elucidating these modality-specific neural mechanisms has important theoretical and practical implications. Theoretically, this study challenges the assumption of a generalized age-related monitoring deficit, proposing instead a dynamic, modality-dependent model of cognitive aging. Practically, because real-world social interactions and clinical cognitive assessments rely heavily on spoken language, identifying the selective vulnerability of the auditory source monitoring network provides a critical, ecologically valid marker. Understanding this “auditory trap” is essential for distinguishing typical cognitive aging from early pathological trajectories, and for optimizing the design of auditory interfaces and interventions for older populations.

## Methods

### Participants

A total of 39 participants were recruited for this study, comprising two distinct age groups. The younger adults included 29 healthy college students (11 males, 18 females; mean age = 19.31 years, SD = 0.23). The older adult group consisted of 10 cognitively normal participants (4 males, 6 females; mean age = 74.60 years, SD = 8.44). Although the sample size for the older cohort is relatively small, it reflects the application of highly stringent inclusion criteria designed to ensure cognitive uniformity and high-quality electrophysiological data. To confirm their cognitive status, older adults were screened using the Mini-Mental State Examination (MMSE) and the Montreal Cognitive Assessment (MoCA), yielding mean scores of 28.11 (SD = 1.23) and 27.26 (SD = 1.15), respectively. All participants were right-handed, native speakers of Mandarin Chinese, with normal or corrected-to-normal vision and normal hearing thresholds. Prior to the experiment, written informed consent was obtained from each participant. The experimental protocol was reviewed and approved by the Ethics Committee of Shenzhen University Medical School (approval no. PN-202500210).

### Stimuli and experimental procedure

**Figure 1:**
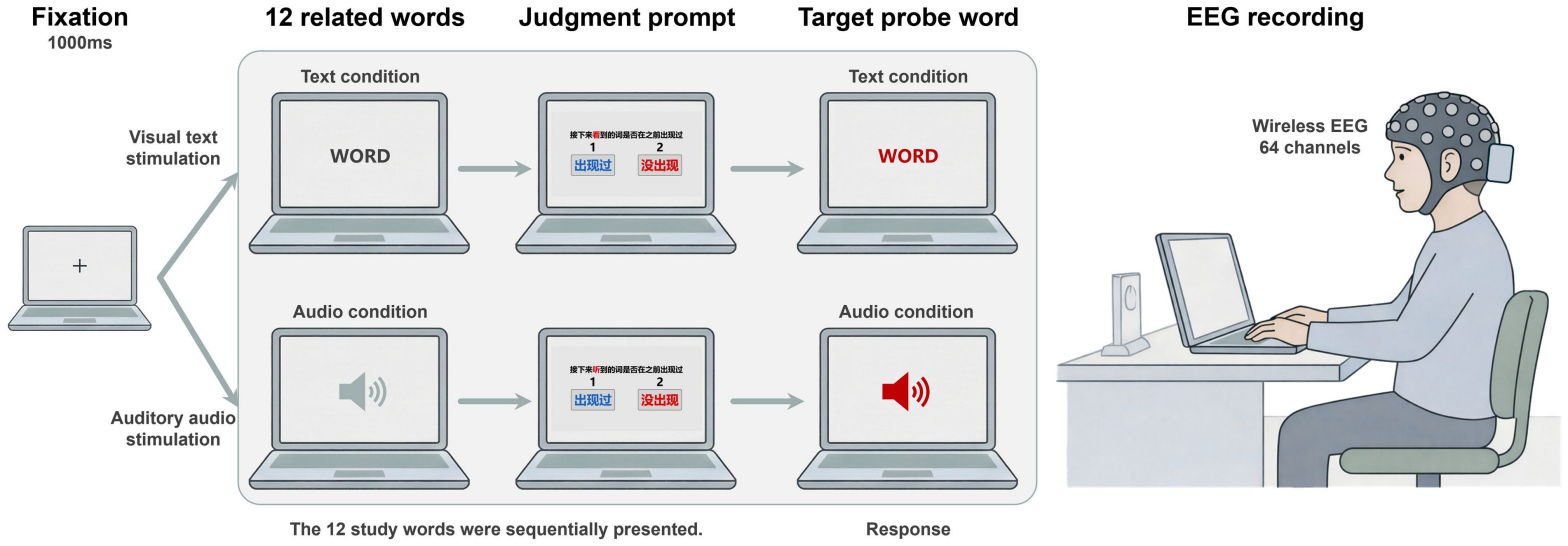
Experimental procedure of the cross-modal DRM memory retrieval task with wireless EEG recording.

The experimental procedure was programmed in MATLAB (R2023a; The MathWorks, Inc.) using the Psychophysics Toolbox (Version 3), as illustrated in Figure 1. Experimental procedure of the cross-modal DRM memory retrieval task with wireless EEG recording. The study employed a modified cross-modal DRM paradigm to investigate the generation and monitoring of false memories under different sensory modalities. The stimulus materials consisted of semantic word lists, with each study trial comprising a list of semantically related associate words followed by a single target probe word.

The experiment was conducted under two modality conditions in a within-subject design:

#### Visual condition

The study words were sequentially presented in the center of a computer screen. Following the presentation of the study list, a visual prompt screen appeared, instructing participants to prepare for the visual recognition judgment. Subsequently, the target probe word was presented on the screen in red font to clearly differentiate it from the study phase.

#### Auditory condition

The 12 study words were presented sequentially via auditory playback through headphones. Following the study list, a corresponding visual prompt screen appeared, instructing participants to prepare for the auditory recognition judgment, after which the auditory probe word was played.

In both conditions, participants were instructed to perform an Old/New recognition task. As indicated by the prompt screens, participants pressed “Key 1” if they believed the probe word had appeared in the preceding study list (“Old”), and “Key 2” if they believed it had not appeared (“New”). The probe words could be “Old” items (previously studied targets) or “Critical Lures” (semantically related words that were never presented). Participants were required to respond as quickly and accurately as possible.

### Behavioral data analysis

Behavioral performance was systematically evaluated across three core outcome metrics: Hit rate (correctly endorsing old items), False Alarm (FA) rate (erroneously endorsing critical lures), and Correct Rejection (CR) rate (successfully rejecting critical lures). Old-lure discrimination capacity was quantified as the difference between the Hit rate and the FA rate.

To rigorously assess the main effects and interactions of age group, sensory modality, and item type on memory susceptibility, we employed a Generalized Linear Mixed-Effects Model (GLMM) with a binomial link function for the binary response data (“Old” vs. “New”). For the temporal dynamics of source monitoring, Reaction Times (RTs) for Hits, FAs, and CRs were log-transformed to satisfy normality assumptions and analyzed using a Linear Mixed-Effects Model (LMM) (Gałecki and Burzykowski, 2013). Both models included Age Group, Modality, and Item Type (or Condition) as fixed effects, alongside random intercepts for participants and items to account for within-subject and stimulus-specific variability.

### EEG data acquisition

Continuous EEG data were acquired during the memory retrieval phase using a NeuralShell W64S system (NeuralEcho Technology Co., Ltd., Beijing, China), a portable, wireless, dry-electrode EEG acquisition device. The system recorded from 64 channels positioned according to the international 10–20 system at a sampling rate of 1,000 Hz. An online reference was used based on the default configuration of the device, and impedances were monitored wirelessly throughout the experiment to ensure optimal signal quality. Before data acquisition, all channels were checked to ensure stable scalp contact and acceptable signal quality according to the manufacturer’s recommended impedance range for dry-electrode recording. Channels showing unstable or excessively noisy signals were readjusted prior to testing. During recording, signal quality was continuously inspected by the experimenter to minimize motion-related and contact-related artifacts.

Traditional wet-electrode EEG systems often require lengthy preparation times (e.g., skin abrasion and gel application) and can physically restrict movement (Liu et al., 2023; Zhang et al., 2025). These factors can induce significant stress and anxiety, particularly in elderly individuals. To maximize ecological validity and minimize participant burden, we specifically utilized this wireless dry-electrode system. This innovative setup allowed for rapid deployment and provided a more comfortable, naturalistic testing experience, thereby ensuring higher compliance and superior data quality in our older adult cohort.

### EEG preprocessing and event-related potential (ERP) analysis

Offline EEG preprocessing was conducted using EEGLAB (Delorme and Makeig, 2004) in MATLAB. Continuous data were band-pass filtered (0.5–30 Hz), segmented into epochs (−200 to 1,000 ms relative to probe onset), and baseline-corrected (−200 to 0 ms). Epochs containing artifacts exceeding an absolute amplitude threshold of ±100 μV were automatically rejected.

Subsequent ERP analyses focused on mean amplitudes extracted from two *a priori* regions of interest (ROIs): the Frontal ROI (FP1, AF3, F1, F3, F5, F7, FC1, FC3, FC5, FPz, AFz, Fz, FCz, FP2, AF4, F2, F4, F6, F8, FC2, FC4, FC6) and the Parietal ROI (Fz, FCz, Cz, CPz, Pz, CP1, CP2, P1, P2). To capture the dynamic neurocognitive processes of memory retrieval, statistical analyses were conducted across two critical temporal windows: an early processing window (300–500 ms), with FN400 effects examined in the Visual condition to index familiarity-related processing and N400-like effects examined in the Auditory condition to index semantic conflict processing (Shayesteh et al., 2020), and a late monitoring window (600–800 ms) to evaluate post-retrieval detail recollection and conflict resolution (P300/LPC effects) (Wang et al., 2022).

Repeated-measures analyses of variance (RM-ANOVA) were conducted on the mean amplitudes extracted from the predefined ROIs. To rigorously account for the unequal sample sizes between the two age groups and to control for potential violations of sphericity, the Greenhouse–Geisser correction was strictly applied to all within-subject effects and interactions. Degrees of freedom and *p*-values were adjusted accordingly where appropriate. Furthermore, partial eta squared ( $\eta_{p}^{2}$ ) was calculated and reported to demonstrate the robustness of the effect sizes independent of the sample size.

## Results

### Descriptive statistics and memory susceptibility

To investigate the dynamic effects of age, sensory modality, and item type on memory retrieval, we employed generalized GLMM for response tendencies and LMM for reaction times (Gałecki and Burzykowski, 2013). Comprehensive descriptive statistics and full model outputs are provided in the Supplementary Tables S1 and S2.

**Figure 2:**
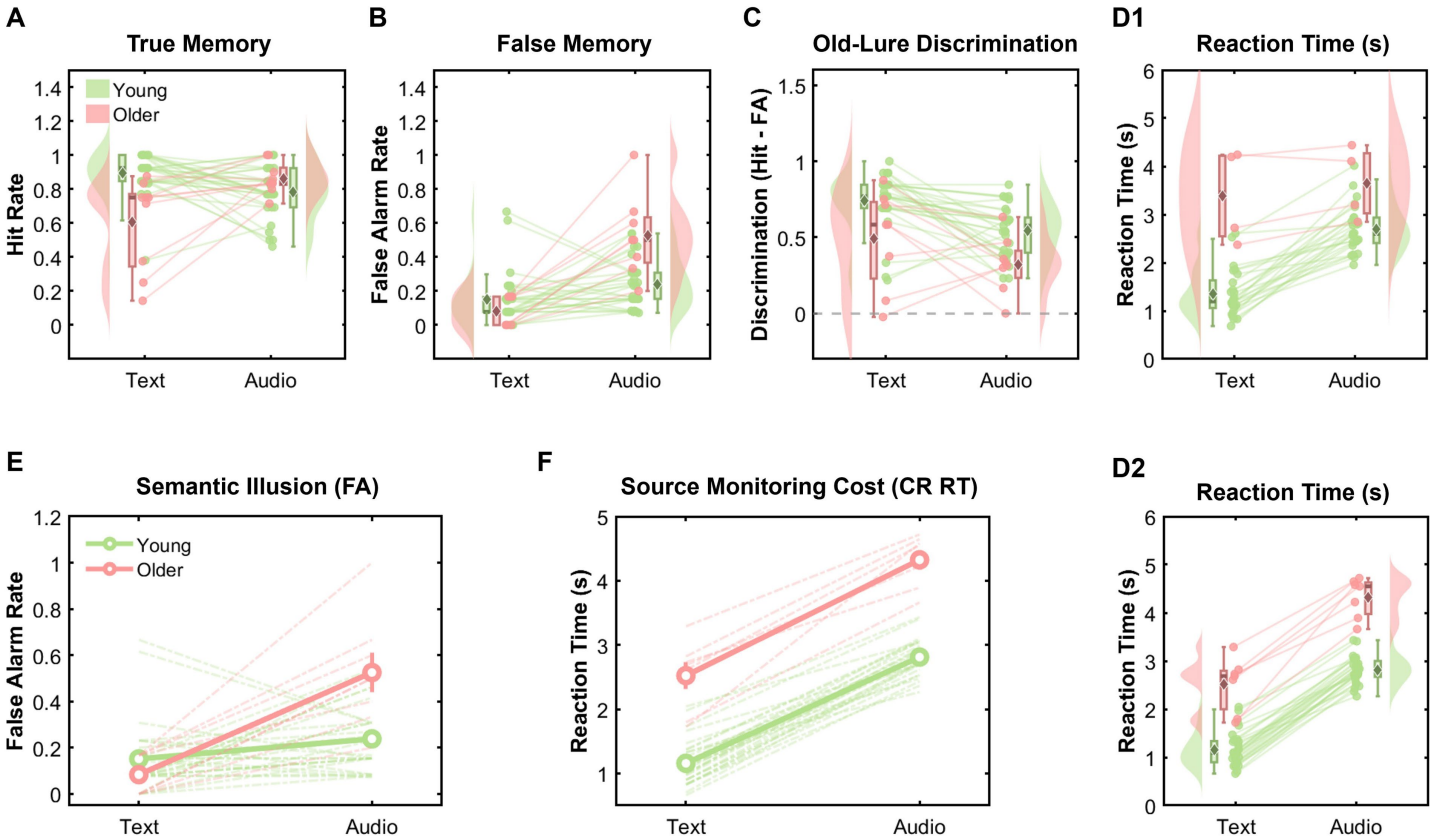
Behavioral results of true and false memory retrieval across sensory conditions. **(A–D2)** Paired raincloud plots display the probability density distributions (half-violins), medians and interquartile ranges (boxplots), and individual participant data (scatter points) for Young (green) and Older (red) adults. Solid lines connect within-subject data across Visual and Auditory conditions. Panels show true memory **(A)**, false memory **(B)**, old-lure discrimination **(C)**, and reaction times for false alarms **(D1)** and correct rejections **(D2)**. **(E,F)** Interaction line plots isolating the semantic illusion rate **(E)** and source monitoring time costs **(F)**. Faint lines represent individual participant trajectories, while bold lines and markers denote group means with standard errors (SEM). The steep upward trajectories of the older adults in the Auditory condition demonstrate a severe breakdown in source monitoring and the massive temporal costs required to successfully reject semantic lures.

The GLMM on the probability of an “old” response revealed a significant main effect of Item Type (*F*(1, 1795) = 91.83, *p* < 0.001), indicating generally higher endorsement rates for old items (Hits) than critical lures (False Alarms). Importantly, a highly significant Group × Item Type interaction (*F*(1, 1795) = 23.43, *p* < 0.001) was observed. As visualized in the data distributions in Figures 2A,B, older adults exhibited a disproportionate increase in false alarms to critical lures compared to younger adults, robustly reflecting age-related source monitoring deficits (McCabe and Smith, 2002).

Crucially, this aging effect was further modulated by encoding modality, evidenced by a significant three-way interaction of Group × Modality × Item Type (*F*(1, 1795) = 5.47, *p* = 0.019). To precisely pinpoint the source of this interaction, Figure 2E isolates the specific trajectories of semantic illusion (FA rate) across groups and conditions. While older adults maintained a remarkably low false alarm rate in the Visual condition (M = 0.14, see Figures 2B,E) comparable to younger adults (M = 0.15), their false memory susceptibility surged dramatically in the Auditory condition (M = 0.49). This steep upward trajectory in older adults (Figure 2E) contrasts sharply with the stable performance of younger adults. Consequently, as shown in the comprehensive raincloud plots (Figure 2C), the old-lure discrimination capacity of older adults plummeted precipitously when transitioning from visual to auditory inputs. This robust interaction suggests that while older adults successfully leveraged visual orthographic cues to suppress false memories, they suffered from a severe breakdown in source monitoring and were particularly susceptible to semantic illusions when relying solely on auditory processing (Baum and Stevenson, 2017; Vallesi et al., 2021).

### Reaction time and monitoring costs

The LMM on log-transformed RTs yielded significant main effects of Group (*F*(1, 1,627) = 112.12, *p* < 0.001) and Modality (*F*(1, 1,627) = 220.82, *p* < 0.001), along with a significant Group × Modality interaction (*F*(1, 1,627) = 31.77, *p* < 0.001). These results confirm general cognitive slowing in older adults, which was disproportionately exacerbated during the auditory task (Rey-Mermet and Gade, 2018).

While the main effects of Condition (Hit, FA, CR) were not significant (*p* = 0.983), a trend-level three-way interaction of Group × Modality × Condition emerged (*F*(2, 1,627) = 2.97, *p* = 0.052). Figure 2F illustrates a possible divergence in response-time patterns across conditions, particularly among older adults under auditory presentation. Descriptive comparisons of Figure 2D1 (Lure-FA) and Figure 2D2 (Lure-CR) suggested that older adults tended to require more time to correctly reject lures (*M* = 4.21 s) than to falsely endorse them (*M* = 3.36 s). This pattern may reflect increased monitoring demands during successful lure rejection, although the marginal significance of the interaction warrants cautious interpretation and further replication. One possible explanation is that familiarity-based responding may have been relatively faster than effortful source-monitoring processes under auditory conditions (Whitney et al., 2008).

### Cross-modal divergence in early familiarity and semantic conflict

**Figure 3:**
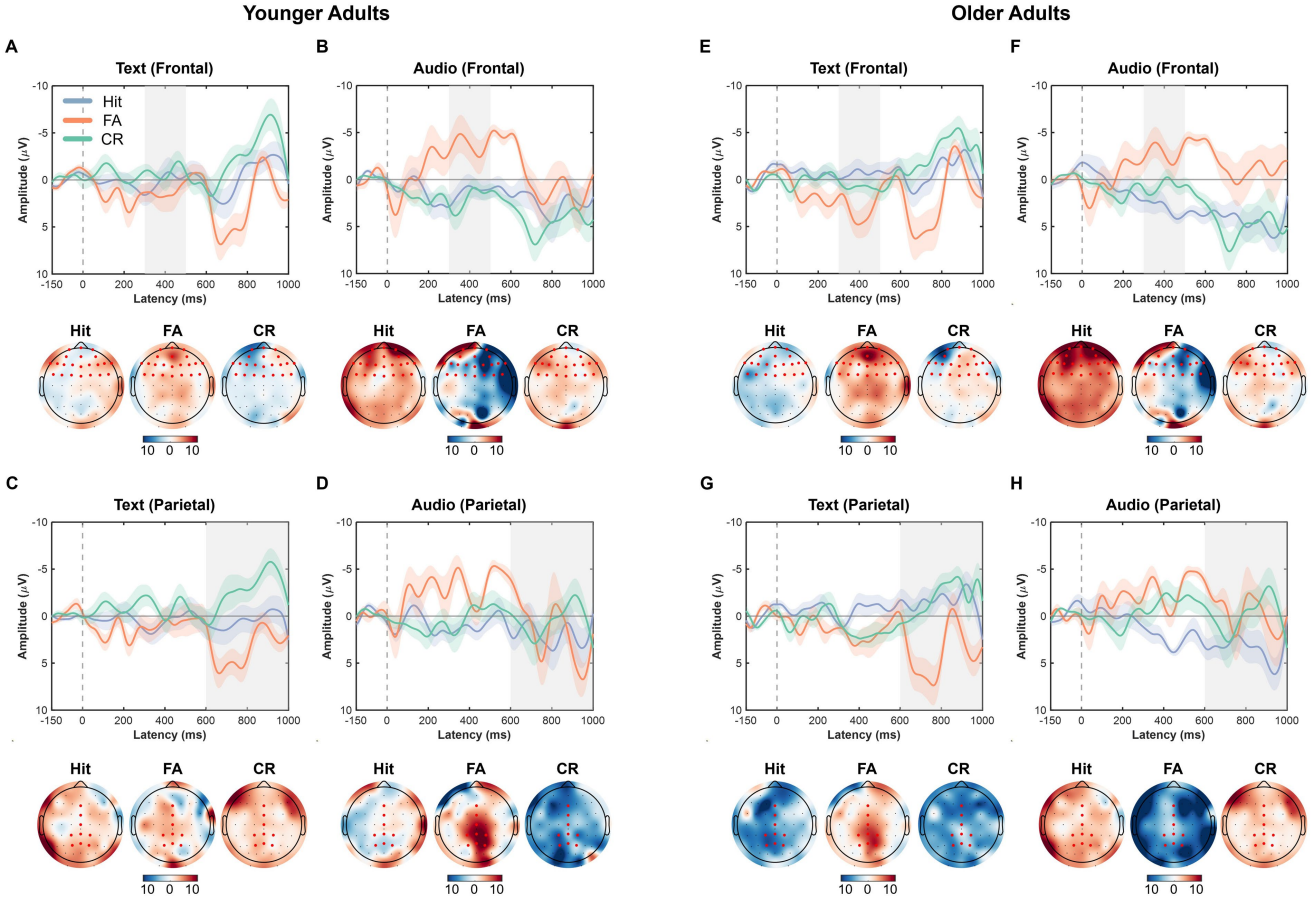
Grand average ERPs and topographical maps during memory retrieval. **(A–H)** Display data for Young and Older adults, respectively. Shaded grey areas indicate the early processing (300–500 ms) and late monitoring (600–800 ms) windows. Topographic maps below each waveform illustrate the spatial distribution of mean amplitudes within these windows, with red dots denoting the Frontal and Parietal ROI electrodes. Note the striking modality-dependent divergence: lures in the Visual condition elicit a frontal positivity, whereas Auditory lures (FA, orange lines) trigger a massive early frontocentral negativity (N400-like effect). Crucially, older adults exhibit severely disorganized waveforms and diffuse topographical patterns during late auditory parietal monitoring **(H)**, contrasting with the preserved differentiation in younger adults **(D)**. Shaded bands represent the standard error of the mean (SEM).

To elucidate the neural mechanisms underlying the profound behavioral susceptibility to auditory semantic illusions, we analyzed the event-related potentials (ERPs) and their spatial distributions across Frontal and Parietal regions of interest (ROIs). Visual inspection of the grand average waveforms and topographic maps (Figure 3) revealed a striking modality-dependent divergence in early memory retrieval stages (300–500 ms).

In the Visual condition, both Young (Figures 3A,C) and Older adults (Figures 3E,G) exhibited typical familiarity-driven memory signatures. Specifically, within the early 300–500 ms time window (FN400 region), both true memories (Hit) and false alarms to critical lures (FA) elicited more positive-going waveforms compared to correct rejections (CR). This visual observation was robustly supported by a 3-way RM-ANOVA on frontal mean amplitudes (detailed in Supplementary Table S4), which revealed a significant Group × Condition interaction (*F*(2, 74) = 4.24, *p* = 0.024) and a marginally significant three-way Group × Modality × Condition interaction (*F*(2, 74) = 3.00, *p* = 0.065). Follow-up analyses confirmed that in the visual modality, Hit and FA responses were statistically indistinguishable (*p* = 0.764), reflecting a classic FN400 familiarity effect. The accompanying topographic maps (Figures 3A,E) corroborate this, showing a distinct positive spatial distribution over the frontal electrodes for both Hit and FA conditions. This preserved early familiarity effect in older adults perfectly aligns with their relatively intact old-lure discrimination capacity in the visual condition, suggesting that visual orthographic cues successfully support typical conceptual processing regardless of age.

Crucially, this early neural signature was completely inverted in the Auditory condition. Rather than eliciting a familiarity-related positivity, auditory false alarms (FA) evoked a massive early negativity (N400-like effect) across both Frontal and Parietal ROIs in Young (Figures 3B,D) and Older adults (Figures 3F,H). Post-hoc comparisons confirmed this modality-specific inversion, demonstrating that auditory false alarms elicited a significantly more negative-going deflection compared to true memories (*p* = 0.013; Supplementary Table S5). Topographical analysis clearly illustrates this N400-like effect as a concentrated, deep negative focal point over the frontocentral scalp exclusively for the FA condition. This profound negative deflection indicates that auditory lures triggered acute semantic conflict and massive prediction errors early in the processing stream, overriding typical familiarity signals.

### Age-related shifts in late parietal monitoring

To precisely isolate the late positive component (LPC) associated with detail recollection and post-retrieval evaluation, we refined our late parietal analysis to the 600–800 ms time window.

During the visual condition, both age groups successfully recruited late monitoring resources, evidenced by pronounced LPCs in response to false alarms (Yang et al., 2019), supported by strong parietal positivity in the topographic distributions (Figures 3C,G). However, in the highly demanding Auditory condition, clear age-related neurophysiological divergences emerged. As detailed in Supplementary Table S6, the 3-way RM-ANOVA on parietal mean amplitudes revealed a marginally significant Group × Modality × Condition interaction (*F*(2, 74) = 3.20, *p* = 0.055, $\eta_{p}^{2}$ = 0.176). Additionally, a marginally significant Modality × Condition interaction was observed (*F*(2, 74) = 3.03, *p* = 0.063).

These interactions provide direct statistical support for the observed waveforms. Follow-up simple effects analyses (Supplementary Table S7) revealed a striking age-related divergence in processing auditory lures. Under auditory demands, younger adults successfully differentiated false alarms from both true memories (Hit vs. FA: *p* = 0.002) and correct rejections (FA vs. CR: *p* = 0.002), indicating a highly effective and robust late monitoring mechanism that isolates misleading cues (Figure 3D). In stark contrast, this critical neurophysiological differentiation was entirely absent in older adults across all conditions (all *p* > 0.37). As shown in Figure 3H, the late parietal waveforms of older adults in the Auditory condition became highly variable and attenuated, with the neural signatures for true memory (Hit), false alarms (FA), and successful lure rejection (CR) heavily entangled. Rather than a total absence of neural activity, this lack of organized differentiation reflects an inefficient and desynchronized recruitment of late parietal monitoring signals (Cohen and Ridderinkhof, 2013), perfectly mirroring their pronounced behavioral deficit and massive temporal costs in rejecting auditory lures.

## Discussion

The present study provides novel behavioral and electrophysiological evidence elucidating the domain-specific vulnerabilities of source monitoring in cognitive aging. By employing a cross-modal DRM paradigm, we demonstrated that while older adults maintain a remarkably intact capacity to suppress false memories in the Visual condition (McCabe and Smith, 2002), they suffer a reduced performance in old-lure discrimination during auditory processing (Failes et al., 2020). Crucially, our ERP data reveal that this auditory-specific susceptibility is not merely a consequence of passive signal decay, but rather stems from a profound disruption in the neurocognitive dynamics of memory retrieval: an early surge of unresolved semantic conflict (an N400-like effect) followed by reduced recruitment of late parietal monitoring resources (Keating et al., 2017; Oedekoven et al., 2013).

### The auditory trap: modality-specific vulnerability in aging

Behaviorally, our results highlight a severe, modality-dependent source monitoring deficit in older adults. In the Visual condition, older adults exhibited robust old-lure discrimination and remarkably low false alarm rates, comparable to those of younger adults. This suggests that stable orthographic cues provide reliable perceptual details that older adults can successfully leverage to reject semantic illusions (Palmiero et al., 2014). However, transitioning to the Auditory condition precipitated a dramatic surge in false alarms and a disproportionate increase in reaction times for correct rejections. Unlike written text, the auditory stream is transient; phonetic and acoustic details decay rapidly from sensory buffers (Lu et al., 1992). Consequently, older adults, who experience age-related declines in fluid sensory processing (Hong et al., 2024), are forced to over-rely on the extracted “semantic gist” (Abadie and Guette, 2025; Grilli and Sheldon, 2022). When perceptual details fade, the robust semantic activation triggered by the auditory lures overwhelms their monitoring capacity, leading to the observed behavioral collapse.

### Early neural signatures: from familiarity to semantic conflict

The ERP data from the frontal ROI provide striking neurophysiological insights into this modality effect. In the Visual condition, we observed a classic FN400 familiarity effect across both age groups, where true memories (Hits) and false memories (Lure-FAs) elicited similarly positive-going waveforms compared to correct rejections (Shayesteh et al., 2020). This confirms that visual lures successfully bypass the initial semantic gate via conceptual familiarity yet are ultimately caught by subsequent monitoring processes.

Strikingly, this typical familiarity signature was completely inverted in the Auditory condition. Instead of a positive deflection, auditory false alarms elicited a massive early negativity (300–500 ms) characteristic of an N400 semantic conflict effect (Benau et al., 2011). While standard DRM studies utilizing visual text typically observe an FN400 familiarity effect or a reduced N400 for related lures due to semantic priming, the transient nature of auditory speech heavily relies on predictive coding. Consequently, auditory lures that violate strong top-down phonological and semantic expectations can trigger acute prediction errors, thereby reversing the polarity and evoking an enhanced N400-like response (Connolly et al., 2012; Kutas and Federmeier, 2011). This early neurophysiological divergence suggests a fascinating paradox: the older brain does detect an acute semantic incongruity or prediction error when processing auditory lures early in the retrieval stream. However, this early “alarm” signal does not translate into successful behavioral suppression. The intense N400-like negativity likely reflects the frontotemporal semantic network being overwhelmed by competing phonological and semantic representations, creating a cognitive bottleneck that destabilizes subsequent decision-making (Aboud et al., 2023; Vachon and Jolicœur, 2012).

### Age-related shifts in late parietal control

The difficulty in resolving this early auditory semantic conflict becomes unequivocally clear in the late retrieval window (600–800 ms). Successful source monitoring relies heavily on late, top-down frontoparietal control networks, often indexed by the LPC, to engage in effortful post-retrieval evaluation and detail recollection (Shayesteh et al., 2020; Volz et al., 2019; Favila et al., 2018).

Crucially, our ERP data yielded a marginally significant three-way interaction (Group × Modality × Condition), confirming that the breakdown in source monitoring is uniquely tied to the intersection of advanced age and the auditory modality. While our younger cohort demonstrated stable late parietal differentiation between Hits and correct rejections under auditory demands, older adults exhibited heavily entangled parietal waveforms. This inefficient neurophysiological differentiation directly accounts for our reaction time findings. The older adults required massive temporal costs (averaging over 4 s) to correctly reject auditory lures. This prolonged deliberation reflects an effortful struggle to engage depleting parietal resources to overcome the strong semantic pull of the lure. When these cognitive resources are finally overtaxed by the transient nature of auditory inputs, the monitoring system fails to provide a decisive diagnostic signal, forcing older adults to default to heuristic, familiarity-based “old” responses.

### Clinical implications and future directions

These findings extend beyond normal cognitive aging, offering critical insights into the domain-specific neural architecture of auditory processing deficits. The selective vulnerability of the auditory source monitoring network highlighted here may serve as a candidate marker for future studies investigating the transition from typical aging to pathological states, such as Mild Cognitive Impairment (MCI) or early-stage dementia (Jalaei et al., 2019; Thamizhmani et al., 2025). Given that real-world social interaction and clinical assessments heavily rely on spoken language, understanding this “auditory trap” is crucial. Future interventions and diagnostic tools targeting semantic memory or speech perception must account for the disproportionate cognitive load imposed by auditory processing in older populations.

Several limitations warrant consideration. First, the sample size of the older adult group is relatively modest, which may limit the broader generalizability of our findings. This restricted sample size was primarily due to the rigorous cognitive and physical inclusion criteria (i.e., passing both MMSE and MoCA thresholds, requiring strictly normal hearing and vision) combined with the demanding, fatigue-inducing nature of a cross-modal EEG paradigm. Nevertheless, our within-subject design yielded a high number of trials per participant, providing sufficient statistical power to detect the robust, modality-specific neurophysiological divergences (e.g., the N400 inversion and LPC attenuation) reported here. Second, while our scalp EEG provides excellent temporal resolution, future studies utilizing high-density EEG with source localization or fMRI are needed to precisely map the frontoparietal network shifts during this cross-modal task. Finally, extending this paradigm to continuous speech or naturalistic dialogs could further elucidate how older adults navigate semantic illusions in real-world environments.

## Conclusion

Our study suggests that cognitive aging may involve a modality-specific vulnerability to auditory semantic illusions, particularly under conditions requiring effortful source monitoring. While older adults appeared able to use stable visual cues to suppress false memories, transient auditory information may impose greater demands on late parietal monitoring processes. The present electrophysiological findings further support the possibility that auditory false memories in aging arise from an interaction between early semantic familiarity and reduced late-stage monitoring recruitment. By characterizing this cross-modal divergence, the current findings provide a potentially valuable neurocognitive framework for understanding age-related false-memory vulnerability and may offer a promising candidate direction for future investigations into pathological cognitive aging.

## Data Availability

The raw data supporting the conclusions of this article will be made available by the authors, without undue reservation.
