# Supplementary material for: The auditory trap: early semantic conflict and late monitoring breakdown drive false memories in cognitive aging
Source: Front Psychol. 2026 Jul 2;17:1830028. doi: 10.3389/fpsyg.2026.1830028 (PMC13372661; doi:10.3389/fpsyg.2026.1830028)
Supplement: Supplementary file 1 [file Table_1.DOCX]

**Visual DRM Word Lists —Chinese**

**1.睡觉**

床，枕头，被子，打盹，休息，做梦，困倦，躺下，瞌睡，失眠，午休，夜晚，困，安静，疲倦

**2. 水果**

苹果，香蕉，橘子，梨，葡萄，草莓，西瓜，桃子，柚子，樱桃，菠萝，柠檬，芒果，李子，杏子

**3. 颜色**

红，黄，蓝，绿，黑，白，灰，橙，紫，棕，浅色，深色，鲜艳，明亮，暗淡

**4. 家庭**

父亲，母亲，儿子，女儿，爷爷，奶奶，叔叔，阿姨，兄弟，姐妹，父母，亲戚，家人，房子，亲情

**5. 学校**

学生，老师，教室，书本，铅笔，课桌，黑板，粉笔，作业，考试，课本，操场，下课，学习，同学

**6. 食物**

米饭，馒头，面条，包子，饺子，粥，菜，汤，油条，豆浆，鸡蛋，鱼，肉，青菜，水果

**7. 衣服**

裤子，衬衫，外套，裙子，鞋子，袜子，帽子，皮带，手套，围巾，棉袄，毛衣，衣领，纽扣，口袋

**8. 动物**

狗，猫，牛，羊，马，猪，鸡，鸭，鹅，猴，兔，鱼，鸟，熊，老虎

**9. 天气**

晴天，阴天，下雨，刮风，下雪，雷，电，彩虹，寒冷，炎热，凉爽，雾，冰雹，湿润，干燥

**10. 花**

玫瑰，牡丹，菊花，百合，康乃馨，兰花，梅花，桂花，向日葵，荷花，茶花，丁香，郁金香，蒲公英，桃花

**11. 身体**

头，手，脚，眼睛，耳朵，鼻子，嘴，牙齿，胳膊，腿，背，心脏，皮肤，骨头，血液

**12. 交通**

汽车，火车，轮船，飞机，自行车，公交，地铁，出租车，摩托车，马车，道路，桥，隧道，车站，司机

**13. 饮料**

茶，咖啡，牛奶，豆浆，果汁，汽水，啤酒，白酒，红酒，可乐，绿茶，矿泉水，冰水，热水，饮品

**14. 家务**

打扫，做饭，洗衣，拖地，扫地，擦桌子，倒垃圾，收拾，煮饭，浇花，叠衣服，擦窗，整理，晾衣服，做家事

**15. 节日**

春节，中秋，端午，元宵，重阳，清明，国庆，劳动节，圣诞节，元旦，除夕，庙会，拜年，灯会，聚会

**16. 金钱**

人民币，零钱，纸币，硬币，钱包，工资，银行，存款，取款，借钱，还钱，花钱，节约，贵，便宜

**17. 乡村**

农田，稻谷，庄稼，鸡舍，牛棚，土路，农民，村庄，院子，泥土，柴火，炊烟，果园，池塘，树林

**18. 城市**

街道，大楼，商店，超市，马路，工厂，广场，公园，银行，餐馆，红绿灯，出租车，公交，地铁，小区

**19. 医院**

医生，护士，病人，药，病房，手术，病床，打针，输液，挂号，检查，治疗，处方，药房，体温

**20. 运动**

跑步，走路，游泳，打球，踢球，爬山，瑜伽，跳舞，散步，骑车，羽毛球，乒乓球，篮球，体操，锻炼

**21. 植物**

树，草，叶子，根，花，果实，枝，藤，森林，种子，盆栽，庄稼，树木，竹子，灌木

**22. 家具**

桌子，椅子，床，沙发，柜子，凳子，茶几，衣柜，书架，橱柜，抽屉，梳妆台，书桌，餐桌，沙发椅

**23. 厨房**

锅，碗，筷子，勺子，刀，案板，灶台，煤气，油盐，调料，碟子，锅铲，冰箱，电饭锅，炒锅

**24. 早餐**

鸡蛋，牛奶，豆浆，油条，包子，馒头，面包，粥，稀饭，咸菜，花卷，煎饼，米粉，麦片，早点

**25. 亲情**

母爱，父爱，手足，兄弟，姐妹，亲人，关心，照顾，团圆，孝顺，思念，温暖，亲近，情感，家庭

**26. 友谊**

伙伴，同学，同伴，哥们，闺蜜，知己，老同学，老乡，玩伴，兄弟，姐妹，友情，信任，陪伴，关怀

**27. 童年**

玩具，游戏，跳绳，丢沙包，弹珠，捉迷藏，课本，教室，小伙伴，童话，漫画，玩耍，快乐，上学，校园

**28. 晚年**

退休，养老，孙子，孙女，子女，安静，休息，公园，下棋，锻炼，聊天，散步，养生，健康，安心

**29. 音乐**

歌曲，唱歌，舞蹈，乐器，钢琴，吉他，小提琴，笛子，二胡，鼓，曲子，旋律，音符，节奏，合唱

**30. 电影**

电影院，荧幕，演员，导演，剧情，台词，明星，银幕，故事，票，娱乐，戏剧，表演，角色，观众

**Visual DRM Word Lists — English Translation**

**1. Sleep**

bed, pillow, quilt, nap, rest, dream, drowsiness, lie down, doze, insomnia, noon break, night, sleepy, quiet, fatigue

**2. Fruits**

apple, banana, orange, pear, grape, strawberry, watermelon, peach, pomelo, cherry, pineapple, lemon, mango, plum, apricot

**3. Colors**

red, yellow, blue, green, black, white, gray, orange, purple, brown, light color, dark color, bright, vivid, dull

**4. Family**

father, mother, son, daughter, grandfather, grandmother, uncle, aunt, brother, sister, parents, relatives, family members, house, affection

**5. School**

student, teacher, classroom, book, pencil, desk, blackboard, chalk, homework, exam, textbook, playground, class break, study, classmates

**6. Food**

rice, steamed bun, noodles, stuffed bun, dumplings, porridge, vegetables, soup, fried dough sticks, soy milk, egg, fish, meat, greens, fruit

**7. Clothes**

pants, shirt, coat, skirt, shoes, socks, hat, belt, gloves, scarf, cotton jacket, sweater, collar, button, pocket

**8. Animals**

dog, cat, cow, sheep, horse, pig, chicken, duck, goose, monkey, rabbit, fish, bird, bear, tiger

**9. Weather**

sunny day, cloudy day, rain, wind, snow, thunder, lightning, rainbow, cold, heat, coolness, fog, hail, humid, dry

**10. Flowers**

rose, peony, chrysanthemum, lily, carnation, orchid, plum blossom, osmanthus, sunflower, lotus, camellia, lilac, tulip, dandelion, peach blossom

**11. Body**

head, hand, foot, eyes, ears, nose, mouth, teeth, arm, leg, back, heart, skin, bone, blood

**12. Transportation**

car, train, ship, airplane, bicycle, bus, subway, taxi, motorcycle, carriage, road, bridge, tunnel, station, driver

**13. Drinks**

tea, coffee, milk, soy milk, juice, soda, beer, liquor, red wine, cola, green tea, mineral water, ice water, hot water, beverage

**14. Housework**

cleaning, cooking, washing clothes, mopping the floor, sweeping, wiping the table, taking out trash, tidying up, making rice, watering flowers, folding clothes, cleaning windows, organizing, hanging clothes, doing housework

**15. Festivals**

Spring Festival, Mid-Autumn Festival, Dragon Boat Festival, Lantern Festival, Double Ninth Festival, Qingming Festival, National Day, Labor Day, Christmas, New Year’s Day, New Year’s Eve, temple fair, paying New Year visits, lantern show, gathering

**16. Money**

RMB, change, banknotes, coins, wallet, salary, bank, deposit, withdrawal, borrowing money, repaying money, spending money, saving, expensive, cheap

**17. Countryside**

farmland, rice, crops, chicken coop, cowshed, dirt road, farmer, village, courtyard, soil, firewood, cooking smoke, orchard, pond, woods

**18. City**

street, building, shop, supermarket, road, factory, square, park, bank, restaurant, traffic light, taxi, bus, subway, residential community

**19. Hospital**

doctor, nurse, patient, medicine, ward, surgery, hospital bed, injection, infusion, registration, examination, treatment, prescription, pharmacy, body temperature

**20. Sports**

running, walking, swimming, playing ball, football, mountain climbing, yoga, dancing, strolling, cycling, badminton, table tennis, basketball, gymnastics, exercise

**21. Plants**

tree, grass, leaf, root, flower, fruit, branch, vine, forest, seed, potted plant, crops, trees, bamboo, shrub

**22. Furniture**

table, chair, bed, sofa, cabinet, stool, coffee table, wardrobe, bookshelf, cupboard, drawer, dressing table, desk, dining table, armchair

**23. Kitchen**

pot, bowl, chopsticks, spoon, knife, cutting board, stove, gas, oil and salt, seasoning, plate, spatula, refrigerator, rice cooker, wok

**24. Breakfast**

egg, milk, soy milk, fried dough sticks, stuffed bun, steamed bun, bread, porridge, congee, pickles, flower roll, pancake, rice noodles, cereal, breakfast food

**25. Family Affection**

maternal love, paternal love, siblings, brothers, sisters, loved ones, care, looking after, reunion, filial piety, longing, warmth, closeness, emotion, family

**26. Friendship**

partner, classmate, companion, buddy, best friend, confidant, old classmate, fellow townsman, playmate, brother, sister, friendship, trust, companionship, care

**27. Childhood**

toys, games, jump rope, sandbag throwing, marbles, hide-and-seek, textbooks, classroom, little friends, fairy tales, comics, playing, happiness, going to school, campus

**28. Old Age**

retirement, elderly care, grandson, granddaughter, children, quietness, rest, park, chess, exercise, chatting, walking, health preservation, health, peace of mind

**29. Music**

song, singing, dance, musical instrument, piano, guitar, violin, flute, erhu, drum, melody, tune, note, rhythm, chorus

**30. Movies**

cinema, screen, actor, director, plot, lines, celebrity, silver screen, story, ticket, entertainment, drama, performance, character, audience
