# Supplementary material for: The auditory trap: early semantic conflict and late monitoring breakdown drive false memories in cognitive aging
Source: Front Psychol. 2026 Jul 2;17:1830028. doi: 10.3389/fpsyg.2026.1830028 (PMC13372661; doi:10.3389/fpsyg.2026.1830028)
Supplement: Supplementary file 2 [file Table_2.DOCX]

**Auditory DRM Word Lists —Chinese**

**41. 眼睛**

视力、镜片、眼镜、视线、光线、黑眼、睫毛、眼泪、眨眼、眼皮、眼神、眼框、眼角、注视、明亮

**42. 耳朵**

听力、耳机、耳环、响声、耳膜、耳孔、安静、音乐、耳语、声音、耳边、聆听、侧耳、耳垢、聋哑

**43. 双手**

拿着、抓住、握手、拍手、抚摸、伸手、提包、推门、写字、手掌、手指、掌心、打字、放下、牵手

**44. 双脚**

走路、跑步、踩地、弯腿、踢球、伸脚、脚底、脚趾、脚跟、鞋子、袜子、脚印、落地、爬坡、登山

**45. 笑容**

开心、快乐、高兴、喜悦、微笑、幽默、幸福、愉快、笑声、轻松、笑话、调皮、开朗、愉悦、爽朗

**46. 哭泣**

悲伤、难过、流泪、痛苦、委屈、安慰、哽咽、抽泣、心酸、感动、沉默、忧愁、悲哀、失落、难受

**47. 记忆**

回忆、想起、往事、怀念、梦境、脑海、照片、经历、故事、过去、想象、记住、遗忘、片段、时间

**48. 健康**

身体、饮食、运动、锻炼、医生、医院、药物、睡眠、休息、检查、走路、体检、养生、心脏、血压

**49. 疼痛**

酸痛、胀痛、头痛、腰痛、手痛、脚痛、针刺、伤口、扭伤、麻木、痛苦、疼感、肌肉、骨头、痉挛

**50. 药物**

药片、胶囊、药方、剂量、药水、药瓶、感冒、退烧、止痛、吃药、打针、治疗、医生、药店、药袋

**51. 海洋**

海浪、沙滩、海边、渔船、贝壳、鱼群、海风、蓝天、岛屿、珊瑚、潜水、海港、海鸥、浪花、船只

**52. 森林**

树林、树木、草地、阳光、空气、山路、鸟儿、虫子、蘑菇、松林、绿叶、树皮、山坡、林中、清香

**53. 高山**

山顶、石头、山路、云雾、树木、山谷、山脚、坡地、山风、山脉、山峰、登山、山泉、岩石、山景

**54. 河流**

溪水、桥梁、河岸、小船、湖泊、波浪、河堤、流动、钓鱼、清水、湿润、波光、水流、漂浮、渡口

**55. 花园**

花草、树木、花坛、泥土、种子、蝴蝶、蜜蜂、阳光、水壶、花盆、桂花、玫瑰、香气、绿色、花香

**56. 厨具**

锅碗、筷子、勺子、菜刀、碟子、锅盖、灶台、调料、油瓶、电炉、汤勺、围裙、碗筷、锅铲、碗盘

**57. 饮食**

米饭、菜肴、汤面、水果、蔬菜、筷子、餐具、味道、咸味、甜味、香气、饱腹、美食、饭菜、餐桌

**58. 时间**

时钟、日历、早晨、夜晚、今天、昨天、明天、现在、未来、过去、分针、秒针、钟表、时光、瞬间

**59. 天气**

晴天、阴天、暴雨、彩虹、乌云、阳光、雷电、气温、寒冷、潮湿、冰雪、微风、酷热、凉爽、霜冻

**60. 衣物**

上衣、裤子、鞋子、袜子、围巾、手套、帽子、毛衣、外套、皮带、布料、裙子、衣架、夹克、棉袄

**61. 交通**
汽车，火车，飞机，轮船，地铁，自行，摩托，出租，巴士，车票，司机，红灯，车站，桥梁，隧道

**62. 职业**
教师，医生，护士，工人，农民，司机，警察，售货，邮递，厨师，记者，经理，秘书，演员，画家

**63. 商店**超市，柜台，收银，货架，顾客，购物，商品，买卖，价格，收据，打折，袋子，付款，收银，结账

**64. 钱币**
钱包，硬币，钞票，零钱，信用，银行，账单，支付，找零，收入，花费，存钱，借钱，贵重，节约

**65. 房间**卧室，客厅，厨房，浴室，沙发，桌椅，窗帘，灯光，电视，地毯，柜子，门锁，墙壁，床铺，书桌

**66. 节庆**礼物，蛋糕，蜡烛，祝福，亲友，聚餐，红包，花束，喜庆，鞭炮，宴会，欢乐，节日，音乐，笑脸

**67. 动物**
狮子，老虎，猴子，大象，熊猫，长颈，斑马，孔雀，狼狗，狐狸，山羊，白兔，鸟儿，海豚，蛇类

**68. 花木**
花朵，树木，草叶，枝条，果实，绿叶，植物，盆栽，阳光，水分，种子，生命，成长，花香，清香

**69. 书籍**
文字，阅读，小说，报纸，杂志，故事，封面，页码，目录，纸张，学习，笔记，摘抄，书架，图书

**70. 科技**
电脑，手机，网络，屏幕，键盘，鼠标，信号，程序，电视，数据，芯片，智能，电子，科技，设备

**Auditory DRM Word Lists — English Translation**

1. **Eyes**vision, lenses, glasses, line of sight, light, black eye, eyelashes, tears, blinking, eyelid, gaze, eye socket, corner of the eye, staring, brightness
2. **Ears**
   hearing, headphones, earrings, sound, eardrum, ear canal, silence, music, whisper, voice, beside the ear, listening, overhearing, earwax, deaf-mute
3. **Hands**
   holding, grabbing, handshake, clapping, touching, reaching out, carrying a bag, pushing a door, writing, palm, fingers, center of the palm, typing, putting down, hand in hand
4. **Feet**
   walking, running, stepping on the ground, bending legs, kicking a ball, stretching feet, sole, toes, heel, shoes, socks, footprints, landing, climbing slopes, mountain climbing
5. **Smile**
   happy, joy, cheerful, delight, smile, humor, happiness, pleasantness, laughter, relaxation, joke, playful, outgoing, enjoyment, hearty
6. **Crying**
   sadness, sorrow, tears, pain, grievance, comfort, choking up, sobbing, bitterness, being moved, silence, melancholy, grief, loss, discomfort
7. **Memory**
   recall, remembering, past events, nostalgia, dreams, mind, photographs, experiences, stories, past, imagination, memorizing, forgetting, fragments, time
8. **Health**
   body, diet, exercise, workout, doctor, hospital, medicine, sleep, rest, examination, walking, physical check-up, healthcare, heart, blood pressure
9. **Pain**
   soreness, swelling pain, headache, back pain, hand pain, foot pain, stabbing sensation, wound, sprain, numbness, suffering, painful feeling, muscles, bones, cramps
10. **Medicine**
    tablet, capsule, prescription, dosage, liquid medicine, medicine bottle, cold, fever reduction, pain relief, taking medicine, injection, treatment, doctor, pharmacy, medicine bag
11. **Ocean**
    waves, beach, seaside, fishing boat, seashells, fish schools, sea breeze, blue sky, island, coral, diving, harbor, seagull, spray, ships
12. **Forest**
    woods, trees, grassland, sunlight, air, mountain path, birds, insects, mushrooms, pine forest, green leaves, bark, hillside, forest interior, fragrance
13. **Mountain**
    mountaintop, rocks, mountain road, mist, trees, valley, foot of the mountain, slope, mountain wind, mountain range, peak, climbing, spring water, stones, mountain scenery
14. **River**
    stream, bridge, riverbank, boat, lake, waves, embankment, flowing, fishing, clear water, humidity, shimmering waves, current, floating, ferry crossing
15. **Garden**
    flowers and plants, trees, flower bed, soil, seeds, butterflies, bees, sunlight, watering can, flowerpot, osmanthus, roses, fragrance, greenery, floral scent
16. **Kitchenware**
    pots and bowls, chopsticks, spoon, kitchen knife, plate, pot lid, stove, seasoning, oil bottle, electric stove, soup ladle, apron, tableware, spatula, dishes
17. **Diet**
    rice, dishes, noodle soup, fruit, vegetables, chopsticks, tableware, taste, salty flavor, sweet flavor, aroma, fullness, delicacy, meals, dining table
18. **Time**
    clock, calendar, morning, night, today, yesterday, tomorrow, present, future, past, minute hand, second hand, timepiece, time, instant
19. **Weather**
    sunny day, cloudy day, heavy rain, rainbow, dark clouds, sunshine, thunder and lightning, temperature, coldness, humidity, ice and snow, breeze, heat, coolness, frost
20. **Clothing**
    top, pants, shoes, socks, scarf, gloves, hat, sweater, coat, belt, fabric, skirt, hanger, jacket, cotton coat
21. **Transportation**
    car, train, airplane, ship, subway, bicycle, motorcycle, taxi, bus, ticket, driver, red light, station, bridge, tunnel
22. **Occupations**
    teacher, doctor, nurse, worker, farmer, driver, police officer, salesperson, postman, chef, journalist, manager, secretary, actor, painter
23. **Store**
    supermarket, counter, cashier, shelf, customer, shopping, goods, trade, price, receipt, discount, bag, payment, checkout, settlement
24. **Currency**
    wallet, coins, banknotes, change, credit, bank, bill, payment, change returned, income, spending, saving money, borrowing money, valuables, thrift
25. **Room**
    bedroom, living room, kitchen, bathroom, sofa, tables and chairs, curtains, lighting, television, carpet, cabinet, door lock, wall, bed, desk
26. **Celebration**
    gift, cake, candles, blessings, relatives and friends, banquet, red envelope, bouquet, festivity, firecrackers, feast, joy, festival, music, smiling face
27. **Animals**
    lion, tiger, monkey, elephant, panda, giraffe, zebra, peacock, wolfdog, fox, goat, white rabbit, birds, dolphin, snakes
28. **Flowers and Trees**
    flowers, trees, grass leaves, branches, fruit, green leaves, plants, potted plants, sunlight, moisture, seeds, life, growth, floral fragrance, fresh scent
29. **Books**
    text, reading, novel, newspaper, magazine, story, cover, page number, contents, paper, study, notes, excerpts, bookshelf, books
30. **Technology**
    computer, mobile phone, internet, screen, keyboard, mouse, signal, program, television, data, chip, intelligence, electronics, technology, equipment
