# Supplementary material for: The auditory trap: early semantic conflict and late monitoring breakdown drive false memories in cognitive aging
Source: Front Psychol. 2026 Jul 2;17:1830028. doi: 10.3389/fpsyg.2026.1830028 (PMC13372661; doi:10.3389/fpsyg.2026.1830028)
Supplement: Supplementary file 3 [file Table_3.DOCX]

**Supplementary Table S1**

**Title:** Descriptive statistics of behavioral performance across age groups and modalities.

| **Group** |  | **Hit Rate** | **FA Rate** | **Discrimination** | **Reaction Time (FA)** | **Reaction Time (CR)** |
| --- | --- | --- | --- | --- | --- | --- |
| **Younger** | text | 0.89 ± 0.12 | 0.15 ± 0.16 | 0.74 ± 0.19 | 1.36 ± 0.49 | 1.16 ± 0.35 |
|  | audio | 0.78 ± 0.15 | 0.24 ± 0.14 | 0.55 ± 0.17 | 2.69 ± 0.47 | 2.81 ± 0.28 |
| **Older** | text | 0.60 ± 0.27 | 0.14 ± 0.18 | 0.47 ± 0.31 | 3.51 ± 0.89 | 2.81 ± 0.80 |
|  | audio | 0.84 ± 0.08 | 0.49 ± 0.26 | 0.36 ± 0.21 | 3.36 ± 0.98 | 4.21 ± 0.49 |

Note: Values are presented as Mean ± Standard Deviation. ‘Discrimination’ is calculated as the difference between Hit Rate and False Alarm (FA) Rate. RT = Reaction Time. ‘Older’ refers to the normal older adults.

**Supplementary Table S2**

**Title:** Type III ANOVA table for the Generalized Linear Mixed-Effects Model (GLMM) on memory susceptibility.

| **Predictors** | $\boldsymbol{F}$ | $\boldsymbol{d}\boldsymbol{f}_{\boldsymbol{num}}\boldsymbol{,d}\boldsymbol{f}_{\boldsymbol{den}}$ | $\boldsymbol{p}$ | **Significance** |
| --- | --- | --- | --- | --- |
| **(Intercept)** | 40.91 | 1, 1795 | < .001 | *** |
| Group | 23.43 | 1, 1795 | < .001 | *** |
| Modality | 2.25 | 1, 1795 | .134 |  |
| Item Type | 91.83 | 1, 1795 | < .001 | *** |
| Group × Modality | 27.26 | 1, 1795 | < .001 | *** |
| Group × Item Type | 23.43 | 1, 1795 | < .001 | *** |
| Modality × Item Type | 3.99 | 1, 1795 | .046 | * |
| **Group × Modality × Item Type** | **5.47** | **1, 1795** | **.019** | ***** |

Note: The dependent variable is the binary response indicating whether the item was judged as “Old” (1) or “New” (0). Group (Younger vs. Older), Modality (Text vs. Audio), and Item Type (Old target vs. Critical lure) were entered as fixed effects. The model includes random intercepts for participants and items. *** *p* < .001, ** *p* < .01, * *p* < .05.

**Supplementary Table S3**

**Title:** Type III ANOVA table for the Linear Mixed-Effects Model (LMM) on reaction times.

| **Predictors** | $\boldsymbol{F}$ | $\boldsymbol{d}\boldsymbol{f}_{\boldsymbol{num}}\boldsymbol{,d}\boldsymbol{f}_{\boldsymbol{den}}$ | $\boldsymbol{p}$ | **Significance** |
| --- | --- | --- | --- | --- |
| **(Intercept)** | 0.59 | 1, 1627 | .443 |  |
| Group | 112.12 | 1, 1627 | < .001 | *** |
| Modality | 220.82 | 1, 1627 | < .001 | *** |
| Item Type | 0.02 | 2, 1627 | .983 |  |
| Group × Modality | 31.77 | 1, 1627 | < .001 | *** |
| Group × Item Type | 1.03 | 2, 1627 | .356 |  |
| Modality × Item Type | 2.05 | 2, 1627 | .130 | * |
| **Group × Modality × Item Type** | **2.97** | **2, 1627** | **.052** | **^** |

Note: The dependent variable is the log-transformed reaction time to satisfy normality assumptions. Group (Younger vs. Older), Modality (Text vs. Audio), and Condition (Hit vs. False Alarm vs. Correct Rejection) were entered as fixed effects. The model includes random intercepts for participants and items. *** *p* < .001, ** *p* < .01, * *p* < .05, ^ *p* < .10 (marginally significant).

**Supplementary Table S4**

**Title:** Type III ANOVA table for the Repeated-Measures ANOVA on early frontal mean amplitudes (300-500 ms).

| **Predictors** | $\boldsymbol{F}$ | $\boldsymbol{d}\boldsymbol{f}_{\boldsymbol{num}}\boldsymbol{,d}\boldsymbol{f}_{\boldsymbol{den}}$ | $\boldsymbol{p}$ | **Significance** |
| --- | --- | --- | --- | --- |
| **(Intercept)** | 2.81 | 1, 37 | 0.115 |  |
| Group | 0.17 | 1, 37 | 0.688 |  |
| Modality | 0.08 | 1, 37 | 0.777 |  |
| Group × Modality | 3.10 | 1, 37 | 0.098 | **^** |
| Condition | 2.52 | 2, 74 | 0.097 | **^** |
| Group × Condition | 4.24 | 2, 74 | 0.024 | * |
| Modality × Condition | 0.72 | 2, 74 | 0.494 |  |
| **Group × Modality × Condition** | **3.00** | 2, 74 | **0.065** | **^** |

Note: The dependent variable is the mean amplitude (*μ*V) in the 300–500 ms time window over the frontal ROI. Group (Younger vs. Older) was entered as a between-subject factor, while Modality (Text vs. Audio) and Condition (Hit vs. False Alarm vs. Correct Rejection) were entered as within-subject factors. The model evaluates the early neurophysiological signatures of familiarity and semantic conflict. *** *p* < 0.001, ** *p* < 0.01, * *p* < 0.05, ^ *p* < 0.10 (marginally significant).

**Supplementary Table S5**

**Title:** Post-hoc simple effects analysis for the frontal ROI (300-500 ms) comparing memory conditions across visual and auditory modalities.

| **Modality** | **Comparison** | **Mean Difference (**$\boldsymbol{\mu V}$**)** | **Std. Error** | $\boldsymbol{p}$ | **Significance** |
| --- | --- | --- | --- | --- | --- |
| Visual (Text) | FA vs. Hit | -1.12 | 1.58 | .764 |  |
|  | CR vs. Hit | -0.74 | 1.66 | .896 |  |
|  | CR vs. FA | 0.37 | 1.39 | .961 |  |
| Auditory (Audio) | **FA vs. Hit** | **-3.32** | **1.02** | **.013** | ***** |
|  | CR vs. Hit | -2.92 | 1.37 | .116 |  |
|  | CR vs. FA | -0.41 | 1.80 | .972 |  |

Note: The table presents pairwise comparisons between true memory (Hit), false memory (FA), and correct rejection (CR) conditions within each sensory modality for the early frontal time window (300-500 ms). Mean difference is calculated as Condition 1 minus Condition 2. * p < .05.

**Supplementary Table S6**

**Title:** Type III ANOVA table for the Repeated-Measures ANOVA on late parietal mean amplitudes (600-800 ms).

| **Predictors** | $\boldsymbol{F}$ | $\boldsymbol{d}\boldsymbol{f}_{\boldsymbol{num}}\boldsymbol{,d}\boldsymbol{f}_{\boldsymbol{den}}$ | $\boldsymbol{p}$ | **Significance** |
| --- | --- | --- | --- | --- |
| **(Intercept)** | 5.12 | 1, 37 | 0.039 | * |
| Group | 3.20 | 1, 37 | 0.094 | ^ |
| Modality | 0.16 | 1, 37 | 0.695 |  |
| Group × Modality | 0.62 | 1, 37 | 0.445 |  |
| Condition | 1.90 | 2, 74 | 0.167 |  |
| Group × Condition | 0.15 | 2, 74 | 0.863 |  |
| Modality × Condition | 3.03 | 2, 74 | 0.063 | ^ |
| Group × Modality × Condition | 3.20 | 2, 74 | 0.055 | ^ |

Note: The dependent variable is the mean amplitude (*μ*V) in the 600–800 ms time window over the parietal ROI, specifically capturing the Late Positive Component (LPC) associated with post-retrieval monitoring. Group (Younger vs. Older) was entered as a between-subject factor, while Modality (Text vs. Audio) and Condition (Hit vs. False Alarm vs. Correct Rejection) were entered as within-subject factors. *** *p* < 0.001, ** *p* < 0.01, * *p* < 0.05, ^ *p* < 0.10 (marginally significant).

**Supplementary Table S7**

**Title:** Post-hoc simple effects analysis for the parietal ROI (600-800 ms) comparing memory conditions across groups in the auditory modality.

| **Group** | **Modality** | **Comparison** | **Mean Difference (**$\boldsymbol{\mu V}$**)** | ***t*-value** | **df** | $\boldsymbol{p}$ | **Significance** |
| --- | --- | --- | --- | --- | --- | --- | --- |
| Younger | Auditory | **FA vs. CR** | **-2.63** | **-4.17** | **28** | **.002** | ****** |
|  |  | **Hit vs. FA** | **5.12** | **4.18** | **28** | **.002** | ****** |
|  |  | Hit vs. CR | 2.49 | 1.75 | 28 | .108 |  |
| Older | Auditory | Hit vs. CR | 0.98 | 0.79 | 9 | .473 |  |
|  |  | FA vs. CR | 0.03 | 0.02 | 9 | .984 |  |
|  |  | Hit vs. FA | 0.95 | 1.00 | 9 | .373 |  |

Note: The table presents manual post-hoc paired t-tests comparing true memory (Hit), false memory (FA), and correct rejection (CR) conditions within the auditory modality for both younger and older adults in the late parietal time window (600-800 ms). Mean difference is calculated as Condition 1 minus Condition 2. p < .01.
